# Development and Evaluation of Physiologically Based Pharmacokinetic (PBPK) Models to Investigate the Effect of CYP2D6 Polymorphism on Metoclopramide Systemic Exposure

**DOI:** 10.3390/ph19071105

**Published:** 2026-07-17

**Authors:** Iqra Shahzad, Ammara Zamir, Muhammad Fawad Rasool, Amer S. Alali, Iltaf Hussain, Faleh Alqahtani

**Affiliations:** 1 Department of Pharmacy Practice, Faculty of Pharmacy, Bahhaudin Zakariya University, Multan 60800, Pakistan; rphiqrashahzad0595@gmail.com (I.S.); ammarazamir20@gmail.com (A.Z.); 2 Department of Pharmaceutics, College of Pharmacy, Prince Sattam Bin Abdulaziz University, Al-Kharj 11942, Saudi Arabia; a.alali@psau.edu.sa; 3 Center for Drug Safety and Policy, Xi’an Jiaotong University, Xi’an 710049, China; iltafhussain@stu.xjtu.edu.cn; 4 Department of Pharmacology and Toxicology, College of Pharmacy, King Saud University, Riyadh 11451, Saudi Arabia

**Keywords:** metoclopramide, PBPK, polymorphism, CYP2D6

## Abstract

**Background:** Physiologically based pharmacokinetic (PBPK) modeling is a mechanistic tool used to predict how a drug moves through the body by incorporating real human physiology, including organ sizes, blood flows, tissue compositions, and enzyme activities. It has been widely employed to estimate drug exposure in different populations with organ impairment, genotype variabilities, and physiological variations. Metoclopramide is an antiemetic and prokinetic agent that is subject to CYP2D6 polymorphism. The study aims to develop PBPK models for several CYP2D6 variants to predict changes in the pharmacokinetic (PK) behavior of metoclopramide. **Methods**: To conduct this study, a literature review was conducted, and the retrieved physicochemical, biochemical, and PK data were integrated into PK-Sim to develop a PBPK model. Initially, a non-genotype-specific model was developed and extrapolated to genotype-based models. The models were verified using a Visual Predicted Check (VPC), mean predicted-to-observed ratio (R_pre/obs_) values, and mean relative deviation (MRD). **Results:** The simulated profiles were aligned with the reported data, and all the predicted and observed PK parameters were comparable, as the R_pre/obs_ values were within the 0.5–2 range and MRD values were <2. Moreover, an increasing trend in AUC_0–∞_ was observed across CYP2D6*wt/*wt, CYP2D6*wt/*10, CYP2D6*10/*10, and CYP2D6*5/*10, with approximately 1.63-, 2.64-, and 2.88-fold increases compared with the CYP2D6*wt/*wt genotype. **Conclusions**: The models have adequately estimated the PK behavior of metoclopramide across different CYP2D6 variants. These models might be helpful for populations with diverse CYP2D6 genotypes in dose optimization.

## 1. Introduction

Physiologically based pharmacokinetic (PBPK) modeling is a reliable mechanistic tool for estimating drug pharmacokinetic (PK) variability across distinct populations using drug-related physicochemical and biochemical data, along with human-specific physiological parameters [1]. It enables the quantification of absorption, distribution, metabolism, and excretion (ADME) processes by explicitly representing underlying biological systems, thus facilitating informed dose selection in special populations [2]. The United States Food and Drug Administration (US FDA) endorses different modeling and simulation approaches for dose individualization, risk assessment, and regulatory decision-making to reduce reliance on in vivo clinical trials. The amalgamation of PBPK modeling and in vitro–in vivo extrapolation (IVIVE) in drug development enables a shift away from simple empirical methods toward a comprehensive, mechanistic, and dynamic simulation framework [3].

PBPK modeling represents the human body as a multicompartmental system interconnected through blood circulation, and combines physicochemical, physiological, and PK data, which makes it a reliable and decisive tool for individualized pharmacotherapy [4]. Interindividual variations in drug PKs, which can significantly alter systemic exposure, are associated with impaired organ function, age, genetic variabilities, and physiological status. By adjusting physiological and biochemical parameters, PBPK modeling provides a foundation for simulating complex clinical scenarios where PK data is limited or difficult to obtain [5].

Metoclopramide is a benzamide-based prokinetic agent that has been used to treat various gastrointestinal tract (GIT) disorders for over thirty years [6]. Firstly, it was introduced by Justin Bescanon in 1960 and subsequently approved for its clinical use by the FDA in 1980 [7]. It shows a dual mechanism of action, acting on both the central and enteric nervous systems to exhibit its antiemetic and prokinetic properties, respectively. For antiemetic effects, it antagonizes dopamine D_2_ receptors in the chemoreceptor trigger zone (CTZ) of the brain, thus inhibiting emetic signals by suppressing dopamine-mediated stimulation to the vomiting center. Moreover, prokinetic action is achieved by inhibiting peripheral dopamine D_2_ and stimulating serotonin 5-HT_4_ receptors in the GIT. This special combination of agonism and antagonism increases acetylcholine release, leading to relaxation of the esophageal sphincter, decreased gastric emptying time, and enhanced intestinal motility [8,9]. Based on the development of tardive dyskinesia (TD), the FDA issued a black box warning in 2009 against prolonged use of metoclopramide [6].

The Biopharmaceutics Classification System (BCS) categorizes metoclopramide as a class III drug because of its higher solubility and lower permeability [10]. After oral administration, it is quickly absorbed, exhibiting a bioavailability of around 80%, and its extensive tissue distribution is shown by a higher apparent volume of distribution, i.e., 3.5 L/kg. It is metabolized in the liver via oxidation and conjugation pathways, where cytochrome P450 2D6 (CYP2D6) plays a key role in its biotransformation. Approximately 20–30% of metoclopramide is excreted through the kidneys in its parent form, whereas the rest of the dose undergoes metabolic transformation before elimination [7].

In 1970, the interindividual variability in CYP2D6 activity was first reported, and since then it has been a major focus of clinical research. Nevertheless, CYP2D6 comprises only 2% of all CYP enzymes, but about 20–25% of clinically approved drugs are primarily metabolized by this enzyme [11]. To date, 149 CYP2D6 variant alleles from CYP2D6*1B to CYP2D6*149 have been discovered, of which only nine alleles, i.e., *1, *2, *3, *4, *5, *6, *10, *17, and *41, make up almost 95% of CYP2D6 diplotypes. CYP2D6*1 and CYP2D6*2 are fully functional alleles; CYP2D6*10, CYP2D6*17, and CYP2D6*41 are reduced functional alleles, whereas CYP2D6*3, CYP2D6*4, CYP2D6*5, and CYP2D6*6 are categorized as non-functional alleles [12]. CYP2D6 variants are classified into four phenotypes, including ultrarapid metabolizers (UMs), extensive metabolizers (EMs), intermediate metabolizers (IMs), and poor metabolizers (PMs). Its polymorphism is linked with enzyme metabolizing activity, which directly affects drug systemic disposition in the human body [13].

Genetic polymorphism in CYP2D6 significantly affects the PK properties of metoclopramide, as variant alleles with extensive and reduced enzymatic activities may cause subtherapeutic and toxic effects, respectively [14]. A case study has reported an acute dystonic reaction in two patients with homozygous CYP2D6 inactive alleles, i.e., CYP2D6*4/*4 and CYP2D6*4/*5, after receiving metoclopramide [15]. Another case study has also investigated the acute side effects of metoclopramide in a patient with poor metabolizing alleles of CYP2D6 [16]. Similarly, a controlled clinical trial has documented altered metoclopramide PK effects in healthy subjects having different CYP2D6 genotypes [17]. The FDA “Table of Pharmacogenomic Biomarkers” lists metoclopramide, and its approved label states that poor metabolizers of CYP2D6 exhibit slower elimination and a higher risk of adverse events [18]. This regulatory guideline provides clinical evidence of genotype-based dosage modification for metoclopramide.

Over the past few decades, the PBPK modeling approach has been adopted to predict systemic disposition for multiple drugs in several genotype groups, including metoprolol, irbesartan, codeine, and flurbiprofen [1,12,13,19]. Several clinical PK studies have reported the influence of CYP2D6 polymorphism on metoclopramide exposure [17,20]. Furthermore, a conference abstract has reported a PBPK model of metoclopramide with limited methodological details, and it lacks a reproducible mechanistic framework [21]. Therefore, a gap exists regarding the mechanistic characterization and quantitative prediction of genotype-dependent variability in metoclopramide PKs. The primary rationale of the present study is not merely to confirm the association between CYP2D6 genotypes and drug exposure but to develop and evaluate a mechanistic PBPK model that describes metoclopramide disposition across multiple CYP2D6 variants. The objective of this study is to develop and evaluate PBPK models of metoclopramide by integrating CYP2D6-based metabolism to characterize genotype-driven differences in PK parameters, including area under the concentration–time curve from time 0 to infinity (AUC_0–∞_), maximum plasma concentration (C_max_), and clearance (CL). This model-based framework facilitates the understanding and use of pharmacogenomic data for metoclopramide in clinical practice, offering a quantitative tool to promote the paradigm of precise medicine, i.e., delivering the right dose of the right drug to the right patient.

## 2. Results

### 2.1. Development and Verification of Non-Genotype-Specific PBPK Model

**Figure 1:**
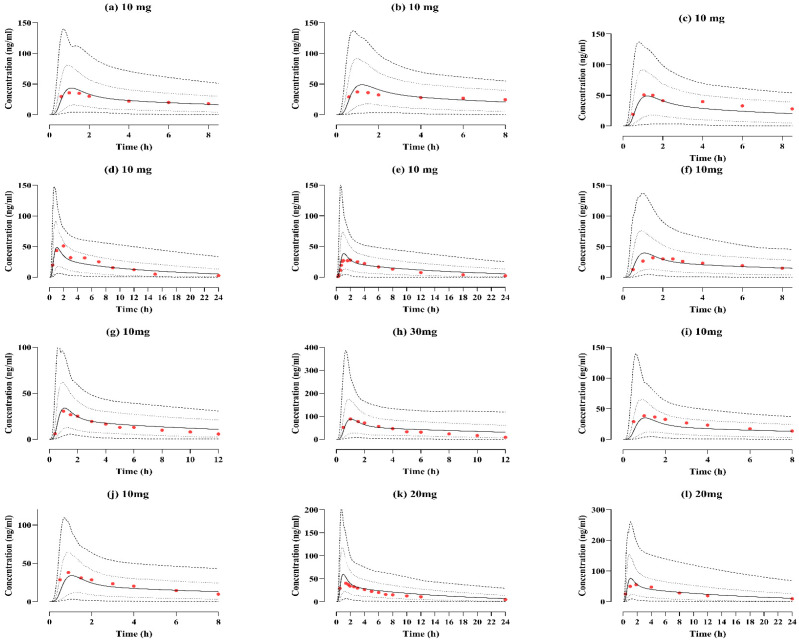
Simulated and observed plasma concentration–time profiles of metoclopramide in a non-genotype-specific adult population following 10–30 mg oral administration. Red dots and solid lines depict reported and predicted data, whereas the 5th–95th centiles and minimum and maximum concentrations are represented by dotted and dashed lines, respectively.

**Figure 2:**
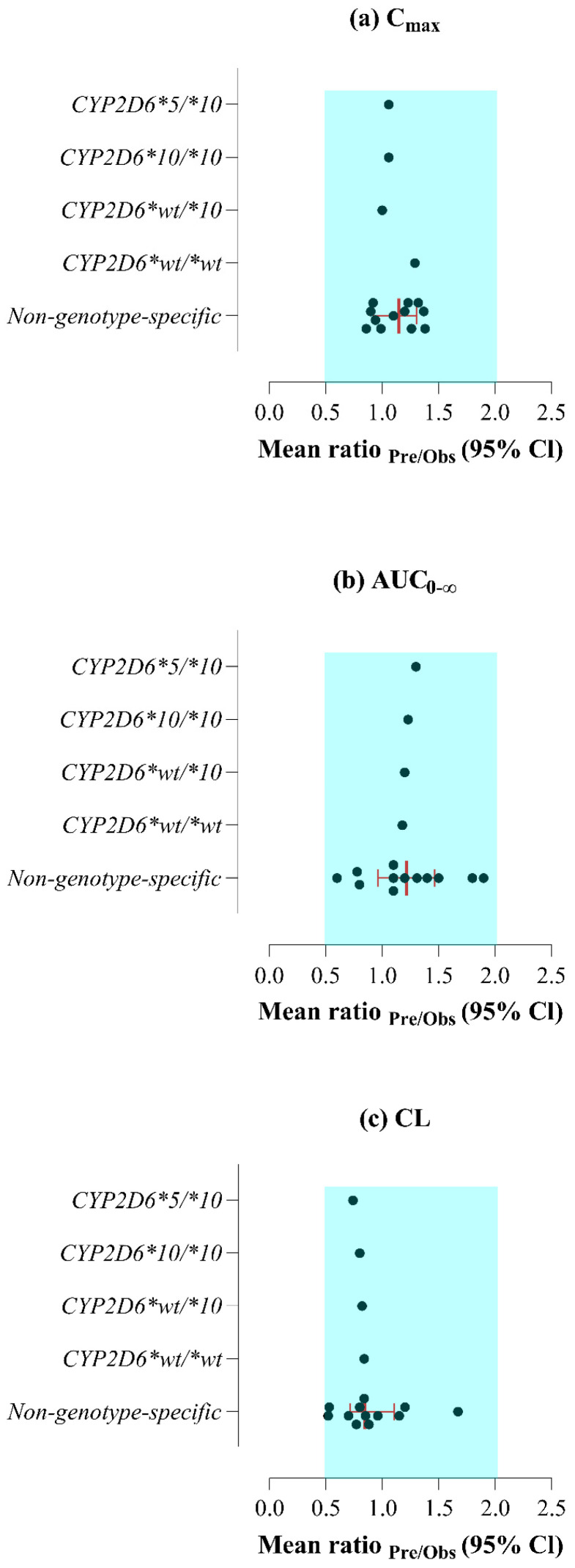
Mean R_pre/obs_ for PK endpoints, including C_max_, AUC_0–∞_, and CL in genotype-non-specific and genotype-specific populations following oral administration of metoclopramide. C_max_: maximum plasma concentration, AUC_0–∞_: area under the plasma concentration time curve from time 0 to infinity, CL: clearance.

Overall, 12 clinical profiles were collected through the literature screening, of which four were used in model development and calibration, while the remaining eight were employed for model verification. The alignment between the estimated and reported plasma concentration profiles of metoclopramide at a dose of 10–30 mg is represented in Figure 1. When compared with the 5th–95th percentiles, the observed data were consistent with predicted graphs and depicted close agreement with the arithmetic mean. The R_pre/obs_ values of C_max_, AUC_0–∞_, and CL for all profiles were within an allowable range, i.e., 0.5–2-fold error. The mean R_pre/obs_ values for all PK parameters are graphically illustrated in Figure 2. The MRD values of all 12 PK profiles were within the range of 1.21–1.65; details are presented in Table 1.

### 2.2. Development and Verification of Genotype-Specific PBPK Model

**Figure 3:**
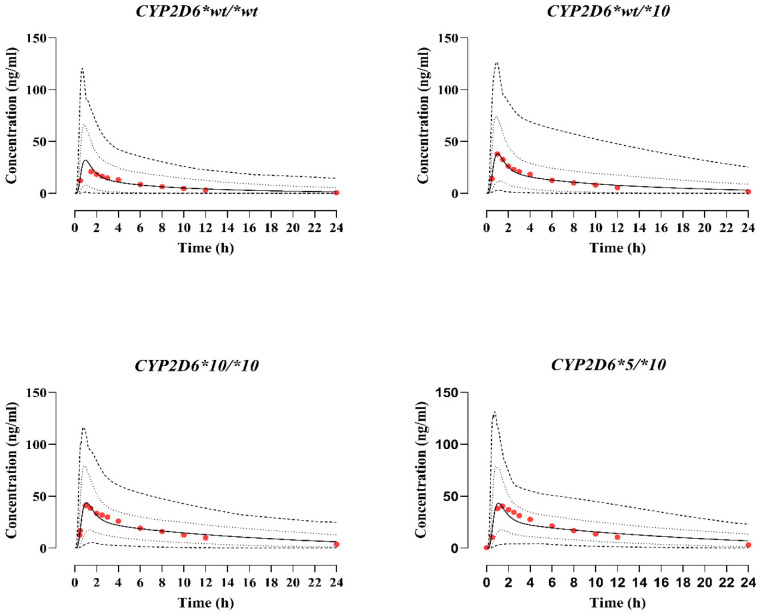
Simulated and observed plasma concentration–time profiles of metoclopramide for populations with different CYP2D6 genotypes, i.e., CYP2D6*wt/*wt, CYP2D6*wt/*10, CYP2D6*10/*10, CYP2D6*5/*10, following 10 mg oral administration. Red dots and solid lines depict reported and predicted data, whereas the 5th–95th centiles and minimum and maximum concentrations are represented by dotted and dashed lines, respectively.

**Figure 4:**
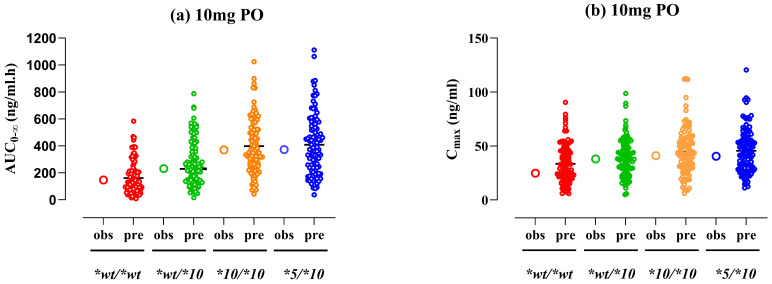
Comparison between the PBPK model’s predicted and clinically observed values of AUC_0_*_–_*_∞_ (**a**) and C_max_ (**b**) for metoclopramide across different CYP2D6 genotypes (CYP2D6*wt/*wt, CYP2D6*wt/*10, CYP2D6*10/*10, CYP2D6*5/*10).

Following the development of a non-genotype model, genotype-based models were extrapolated by keeping all drug-related and ADME parameters fixed to the values applied in the base model, except K_cat_. The simulated profiles of all genotype groups, including CYP2D6*wt/*wt, CYP2D6*wt/*10, CYP2D6*10/*10, and CYP2D6*5/*10, were in concordance with documented profiles. The VPCs for these profiles are displayed in Figure 3, along with 5th–95th percentiles. A comparison of the predicted and observed AUC_0–∞_ and C_max_ is presented in Figure 4 via a strip plot, which depicts genotype-based variability in drug exposure and maximal plasma concentration, confirming the predictive performance of the developed models. An increasing trend in AUC_0–∞_ was observed across CYP2D6*wt/*wt, CYP2D6*wt/*10, CYP2D6*10/*10, and CYP2D6*5/*10, with approximately 1.63-, 2.64-, and 2.88-fold increases compared with the CYP2D6*wt/*wt genotype, while an inverse pattern was recorded in CL for these groups. Additionally, a goodness-of-fit plot was developed and is presented in Supplementary Figure S2 to compare clinical plasma concentration datasets against predicted values across CYP2D6*wt/*wt, CYP2D6*wt/*10, CYP2D6*10/*10, and CYP2D6*5/*10. This plot confirms the predicted ability of this PBPK model, as all points are closely clustered around the line of identity and within the 2-fold range. The residual time graph was generated by calculating the residuals as the log difference between the observed and predicted plasma concentrations and plotting them against time. As the residuals are clustered closely around the zero line, suggesting the model has accurately predicted the ADME (Supplementary Figure S3).

The MRD values for CYP2D6*wt/*wt, CYP2D6*wt/*10, CYP2D6*10/*10, and CYP2D6*5/*10 were 1.31, 1.26, 1.21, and 1.36, respectively. The details of R_pre/obs_ values for C_max_, AUC_0–∞_, and CL across all genotype groups are provided in Table 1, and their graphical illustrations are given in Figure 2.

### 2.3. Assessment of Drug Exposure

**Figure 5:**
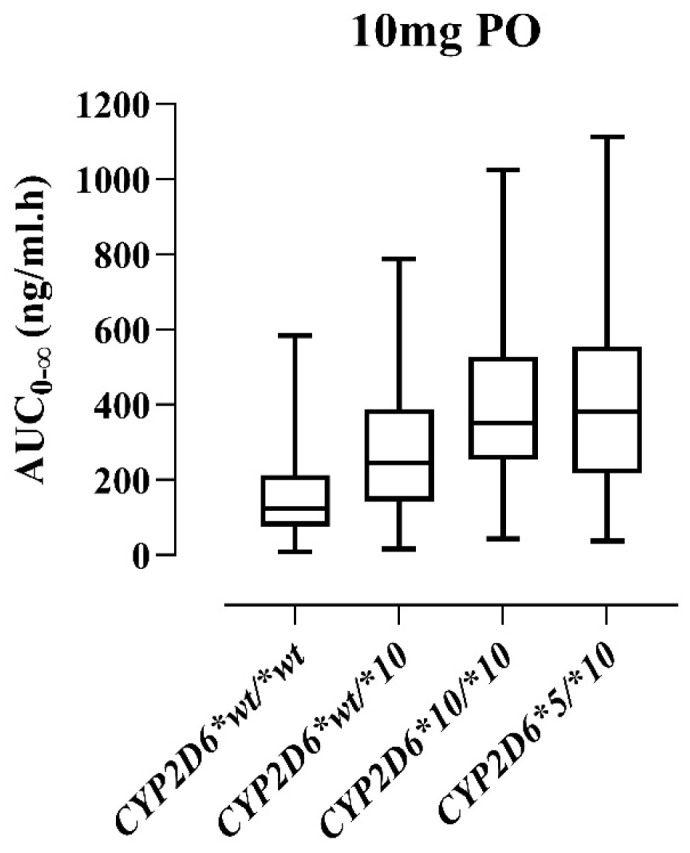
Visual depiction of genotype-based variation in metoclopramide exposure following a 10 mg PO dose by employing box–whisker plots.

The differences in systemic exposure of metoclopramide following a PO dose of 10 mg across CYP2D6*wt/*wt, CYP2D6*wt/*10, CYP2D6*10/*10, and CYP2D6*5/*10 are illustrated in Figure 5. The figure demonstrates a clear increase in drug exposure across CYP2D6*wt/*wt, CYP2D6*wt/*10, CYP2D6*10/*10, and CYP2D6*5/*10, confirming the need for close monitoring of side effects among the population having poor functional alleles of CYP2D6.

## 3. Discussion

Genetic polymorphism of drug-metabolizing enzymes is a major determinant of interindividual variability in therapeutic outcomes, as the metabolic capacities of enzymes play a critical role in influencing a drug’s disposition in the human body [19]. The effect of the genetic variabilities of various enzymes and transporters on the PKs and pharmacodynamics (PDs) of several drugs has been reported in multiple studies [22,23]. Analogous to the different GIT-related drugs, including pantoprazole, esomeprazole, and nortriptyline, metoclopramide is also subject to genetic polymorphism, which affects its PKs and PDs [17,24]. Camilleri et al. documented that the rate and severity of metoclopramide-related side effects varied for different CYP2D6 genetic variants, indicating that different genotypes have altered metabolic capacities [25]. These findings highlight the need for genotype-informed PBPK modeling to elucidate metoclopramide disposition in the context of CYP2D6 variability. Over the last few decades, PBPK modeling has played a substantial role in individualized dose tailoring strategies based on genetic variability, drug–drug interactions (DDIs), age, and diseases (renal and hepatic impairment) [3,19,26].

In the current study, genotype-based PBPK models were developed for metoclopramide to predict PK alterations across four genotype groups (CYP2D6*wt/*wt, CYP2D6*wt/*10, CYP2D6*10/*10, CYP2D6*5/*10). The metabolites of metoclopramide were not considered in this PBPK model construction, as they lack pharmacological activity [27]. The model was developed using a middle-out strategy by incorporating mechanistic knowledge of drug disposition and clinical PK data. The model was verified using the R_pre/obs_, which evaluates its predictive performance using individual PK parameters (AUC_0–∞_, C_max_, CL), and MRD was employed to measure the average fold error between estimated and recorded concentrations over the whole dataset.

According to a study, the primary metabolism of metoclopramide is catalyzed by CYP2D6, with additional pathways including oxidation by CYP3A4, CYP2C19, and CYP1A2, as well as conjugation; however, their metabolic parameters (K_m_, V_max_, K_cat_) are not reported in the literature [28]. To address uncharacterized metabolic routes, the PK-Sim-optimized specific hepatic clearance was integrated into the model. The AS system, initially developed by Gaedigk et al. and later implemented by the CPIC, assigns numerical values to CYP2D6 alleles for translating relative genotypes into phenotypes [29,30]. The adjustment of enzyme kinetic parameters (K_cat_, Cl_int_) based on the AS shows a linear relationship between AS and enzyme activity, which is not always justified due to the involvement of several non-genetic factors, including DDIs and physiological characteristics [31]. Therefore, several studies have developed genotype-based PBPK models by implementing a parameter identification approach [11,13,32]. Following those studies, this research initially developed a non-genotype-specific model and then optimized K_cat_ values for each genotype group, i.e., CYP2D6*wt/*wt, CYP2D6*wt/*10, CYP2D6*10/*10, and CYP2D6*5/*10.

In the non-genotype-specific model, all observed clinical data were comparable to simulated profiles and showed robust agreement with the 5th–95th percentiles, arithmetic mean, and the minimum and maximum plasma concentrations. Additionally, by comparing the estimated and observed PK parameters, including AUC_0–∞_, C_max_, and CL, the predictive ability of this model was assessed (Table 1). The mean estimated C_max_ following PO doses of 10–30 mg was 48.46 ng/mL, which was analogous to the mean observed value, i.e., 43.80 ng/mL. The R_pre/obs_ ranges for AUC_0–∞_ and CL across all included studies were 0.61–1.90 and 0.52–1.67, respectively, both falling within the accepted 0.5–2-fold criteria. The MRD values for all studies were <2, reflecting promising agreement between documented and simulated data.

For all four genotype-specific models, VPCs suggest a clear alignment between observed and simulated profiles, as shown in Figure 3. A progressive increase in the AUC_0–∞_ from CYP2D6*wt/*wt to CYP2D6*5/*10 well captured the impact of less functional alleles (*5 and *10) on drug disposition, suggesting a potential need for dose monitoring (Figure 5). The predicted C_max_ values for CYP2D6*wt/*10 and CYP2D6*10/*10 were 38.10 and 43.59 ng/mL, respectively, and closely matched the observed metrics, i.e., 37.95 and 40.9811 ng/mL. The descending order of predicted CL corresponded to the functionality of genotype alleles, i.e., CYP2D6*wt/*wt>CYP2D6*wt/*10>CYP2D6*10/*10>CYP2D6*5/*10, and all estimates were in agreement with the observed values. The strip plot in Figure 4 describes that the predicted AUC_0–∞_ and C_max_ distributions for 100 individuals accurately captured the documented exposures and maximal plasma concentrations, which fell within the simulated range and were close to the mean. The findings support adequate mechanistic scaling of CYP2D6 activity within this PBPK model framework. The R_pre/obs_ values for C_max,_ AUC_0–∞_, and CL among all genotype groups were within the predefined 0.5–2-fold range. The MRD values were <2 across all genotype-based concentration–time profiles, indicating that the predicted data deviation from observed concentrations was approximately ≤2-fold.

Although a successful PBPK model has been developed, the current study encounters several limitations that must be acknowledged. The PK data of healthy individuals were utilized to design and assess the PBPK model; hence, extrapolation to patient groups with altered physiology should be taken cautiously. Due to a lack of quantified in vitro data, other metabolic pathways were simplified, while the model concentrated on CYP2D6-mediated metabolism. Moreover, the absence of suitable concentration–time profiles in the literature limited the incorporation of groups with ultrarapid and no CYP2D6 activity (ultrarapid and poor metabolizers). In future research, the inclusion of additional genetic variants in CYP2D6 (ultrarapid and poor metabolizers) supported by in vivo clinical PK studies might further improve the predictive performance of the PBPK model.

## 4. Materials and Methods

### 4.1. Modeling Platform

The PBPK model for metoclopramide was developed by a whole-body PBPK simulation software, PK-Sim version 12.1, which was initially programmed by Bayer Technology Services GmbH, Wuppertal, Germany, and is currently managed by the Open Systems Pharmacology community [33]. The concentration–time profiles obtained from extracted publications were digitized by employing the GetData Graph Digitizer software, version 2.26 [34]. Moreover, the PK parameters not reported in relevant clinical studies were computed using non-compartmental analysis (NCA) by a Microsoft Excel Add-ins program, PK Solver [35].

### 4.2. Literature Investigation for Clinical Data

Various medical subject heading (MeSH) terms, including “Pharmacokinetics,” “Pharmacogenomics,” “metoclopramide,” and “Humans,” were used in different research databases to collect clinical studies related to metoclopramide. Four clinical profiles of healthy subjects belonging to different genotype groups, i.e., CYP2D6*wt/*wt, CYP2D6*wt/*10, CYP2D6*10/*10, and CYP2D6*5/*10, were retrieved from a pharmacogenomic study performed in the Korean population. Additionally, nine clinical studies with 12 concentration–time profiles were spotted in the literature, conducted in non-genotype-specific healthy subjects following oral administration of metoclopramide. The model was developed and verified using one-third and two-thirds of the observed profiles, respectively, whereas all datasets were employed in the final evaluation of model predictive performance. The demographic characteristics and dose-related information of the extracted studies are described in Table 2.

### 4.3. Model Structure

Using the middle-out approach, the PBPK model was developed in accordance with the methodologies of well-characterized studies [1,19,45]. The middle-out strategy combines top-down and bottom-up approaches, enabling accurate model scaling based on existing data [46]. The PK-Sim software was selected to conduct this study, in which all body organs are considered as separate compartments, connected via blood circulation. PK-Sim default values of relative expressions for CYP2D6 were applied, imported from various publicly available sources [47]. Virtual individuals were created by using demographic data from published clinical studies to derive physiological characteristics. Metoclopramide, a basic drug with a molecular weight of 299.79 g/mol, mainly binds with alpha-1-acid glycoprotein. The values of various physicochemical properties, including dissociation constant (pK_a)_, solubility, and fraction unbound (f_u_), were integrated as obtained from previous studies without any change, and the lipophilicity (logP) was adjusted within reported ranges, i.e., 1.8–2.667 [48]. PK-Sim-calculated specific intestinal permeability was incorporated; however, due to an overprediction of absorption, it was adjusted from 1 × 10^−5^ cm/min to 0.5 × 10^−5^ cm/min. For specific organ permeability, demonstrating the drug’s distribution characteristics, the estimated value from PK-Sim was used. Rodgers–Rowland and PK-Sim Standard methods were selected to evaluate the intracellular-to-plasma partition coefficient and permeability between the interstitial and cellular spaces, respectively. CYP2D6 is involved in the primary metabolism of metoclopramide; however, its genetic polymorphism results in altered metabolic activity in populations with different genotypes [17]. The metabolism of metoclopramide was described using Michaelis–Menten kinetics based on in vitro data from recombinant CYP2D6. The reported values of the Michaelis–Menten constant (K_m_) and the maximal rate of enzymatic reaction (V_max_) were obtained from a prior study [28]. The turnover number (k_cat_) for the non-genotype-specific PBPK model was calculated and optimized by PK-Sim using the V_max_ value. Subsequently, specific hepatic clearance optimized to 1.8 L/h was integrated to rationalize metabolism not attributed to CYP2D6. Renal plasma clearance, i.e., 0.16 L/h/kg, is a measure of the parent drug’s unaltered excretion from the kidneys. For the oral formulation, the Lint 80 function was applied using a dissolution time of 15 min [10]. The details of the input parameters are mentioned in Table 3. By adopting the published research approach, the Clinical Pharmacogenetics Implementation Consortium (CPIC) activity scoring (AS) was employed to determine the phenotypes of relevant groups, and the non-genotype model was scaled to different CYP2D6 genotype models by optimizing k_cat_ values for each variant group to match the observed profiles using the Monte Carlo method [13,32] (Table 4).

### 4.4. Model Development and Sensitivity Analysis

**Figure 6:**
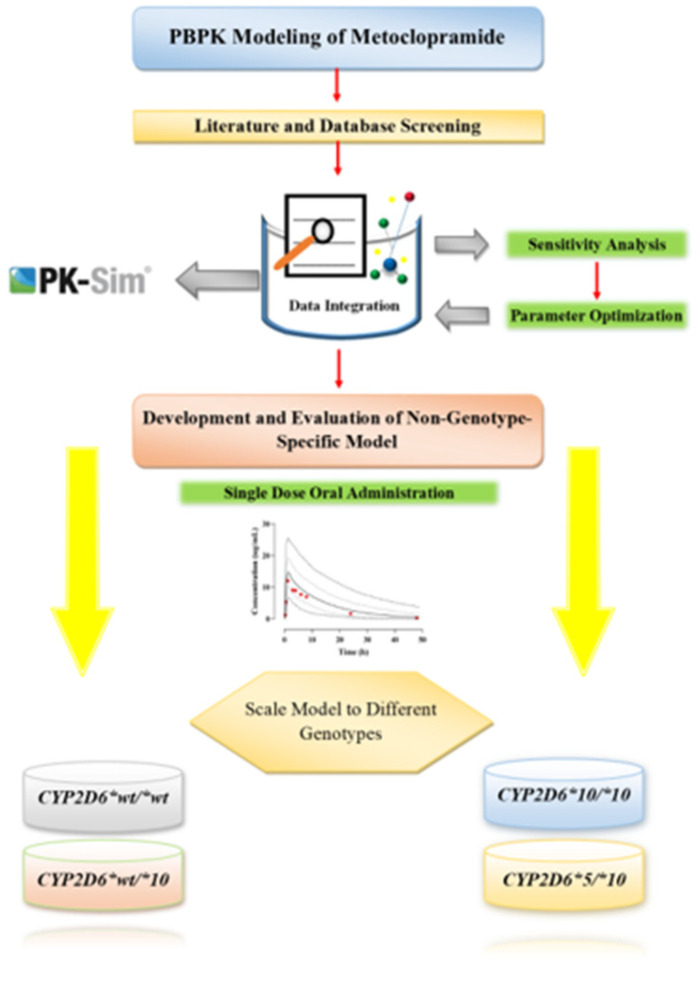
Schematic flowchart for PBPK modeling of metoclopramide. PBPK: physiologically based pharmacokinetic models, CYP: cytochrome P450.

The model development was initiated by performing an extensive literature review to extract physicochemical, PK, and clinical data of metoclopramide. The data were integrated into PK-Sim, and the PBPK model was initially developed for a non-genotype-specific population. A sensitivity analysis was performed in PK-Sim to determine the influence of model parameters on different PK endpoints. The results of the sensitivity analysis are shown in Supplementary Table S1 and Supplementary Figure S1. The sensitive parameters were adjusted manually or by implementing the parameter identification module in PK-Sim. Subsequently, the refined model was evaluated and was considered acceptable once the simulated results aligned with the evaluation criteria. After constructing the PBPK model for non-genotype-specific individuals, the model was extrapolated to populations with different CYP2D6 genotypes by optimizing enzyme activity for each genotype. The model flowchart is illustrated in Figure 6.

### 4.5. Model Verification

Both the visual and numerical approaches were adopted to evaluate the accuracy of the developed PBPK model. Model verification was initiated by creating virtual populations of 100 individuals using demographic characteristics collected from published clinical studies. Using the “visual predictive check (VPC)” strategy, the observed clinical data were assessed alongside the simulated concentration–time profiles by plotting the arithmetic mean, minimum and maximum plasma concentrations, and the 5th–95th percentiles. The Microsoft Excel Add-ins program, PK-Solver, was used to compute the PK parameters, including AUC_0–∞_, C_max_, and CL, for both reported and expected data. Afterward, the model was numerically verified by calculating the “mean predicted-to-observed ratio (R_pre/obs_)”, “fold error”, and “mean relative deviation (MRD)” for all PK profiles using Equations (1)–(3).

(1) $$R=\frac{Predicted\;PK\;parameters}{Observed\;PK\;parameters}$$

(2) $$Fold-error=\frac{Predicted\;values\;of\;parameters}{Observed\;values\;of\;parameters}$$

(3) $$MRD=10^{x},x=\sqrt{\frac{1}{N}\sum_{i=1}^{N}{\left({log}_{10}C_{Pre,i}-{log}_{10}C_{obs,i}\right)}^{2}}$$

For non-genotype-specific profiles, we used R_pre/obs_, along with a 95% CI; however, due to the availability of a single PK profile for each genotype group (CYP2D6*wt/*wt, CYP2D6*wt/*10, CYP2D6*10/*10, CYP2D6*5/*10), R_pre/obs_ was represented as a mean with a range. MRD summarizes the average fold deviation across the entire plasma concentration dataset; an MRD value ≤ 2 is interpreted as indicating adequate model performance [53], whereas the R_pre/obs_ assesses the agreement at individual data points, with values within the 0.5–2.0-fold range considered acceptable [3].

### 4.6. Genotype-Based Variability in Metoclopramide Systemic Exposure

To assess the variability in metoclopramide exposure among CYP2D6*wt/*wt, CYP2D6*wt/*10, CYP2D6*10/*10, and CYP2D6*5/*10 groups, box–whisker plots were generated. Initially, populations of 100 individuals were created for each genotype group, and simulations were run for each group using the administration protocol of a 10 mg PO dose of metoclopramide. The predicted AUC_0–∞_ for all simulated populations was used to generate box–whisker plots, presenting distribution, central tendency, and interpopulation variability. From the PPPM perspective, assessing PBPK-based exposure across all genotype groups, including CYP2D6*wt/*wt, CYP2D6*wt/*10, CYP2D6*10/*10, and CYP2D6*5/*10, may help drive a paradigm shift from traditional reactive dosing strategies to proactive personalized pharmacotherapy.

## 5. Conclusions

The study was conducted to develop and evaluate PBPK models across different variants of CYP2D6, including CYP2D6*wt/*wt, CYP2D6*wt/*10, CYP2D6*10/*10, and CYP2D6*5/*10, to predict the influence of genetic polymorphism on the PK behavior of metoclopramide. All simulated profiles showed adequate agreement with the reported concentrations, and R_pre/obs_ and MRD values were within the accepted 2-fold error range. The PBPK models effectively captured the inverse relationship between metoclopramide systemic exposure and the metabolic activities of CYP2D6 alleles. Within the framework of PPPM, these models may help clinicians to optimize the dose for individuals with different CYP2D6 genotypes to avoid drug-related side effects.

## Figures and Tables

**Table 1:** Results of the development and evaluation of non-genotype-specific and genotype-based PBPK models following oral administration of metoclopramide.

Sr. No.	Genotype	C_max_ (ng/mL)			AUC_0–∞_ (ng/mL·h)			CL(L/h)			MRD
		Obs	Pre	P/O	Obs	Pre	R_pre/obs_	Obs	Pre	R_pre/obs_	
1	Non-genotype-specific	35.79	42.99	1.2	539.23	419.48	0.78	20.0	23.8	1.15	1.13
2	Non-genotype-specific	37.14	46.99	1.26	811.28	493.28	0.6	12.3	20.0	1.67	1.21
3	Non-genotype-specific	50.70	46.88	0.92	604.27	491.12	0.8	16.5	20.0	1.2	1.36
4	Non-genotype-specific	51.03	48.40	0.94	388.33	437.71	1.1	25.7	22.8	0.88	1.50
5	Non-genotype-specific	27.72	38.32	1.38	272.19	401.40	1.4	36.7	25.0	0.7	1.55
6	Non-genotype-specific	31.86	39.46	1.23	309.39	379.67	1.2	32.3	26.3	0.8	1.28
7	Non-genotype-specific	88.81	87.95	0.99	478.29	913.62	1.9	62.73	32.83	0.52	1.65
8	Non-genotype-specific	30.59	33.70	1.1	201.81	380.40	1.8	49.5	26.3	0.53	1.31
9	Non-genotype-specific	38.52	35.02	0.9	287.82	339.27	1.1	34.7	29.4	0.85	1.53
10	Non-genotype-specific	55.17	75.86	1.37	752.48	829.81	1.1	26.5	24.1	0.96	1.49
11	Non-genotype-specific	38.08	32.75	0.86	219.80	340.52	1.5	45.3	38.4	0.84	1.39
12	Non-genotype-specific	40.26	53.21	1.32	410.79	539.52	1.31	48.8	37.7	0.77	1.28
13	CYP2D6*wt/*wt	24.76	32.06	1.29	146.99	173.90	1.18	68.03	57.50	0.84	1.31
14	CYP2D6*wt/*10	37.95	38.10	1	231.62	282.59	1.2	43.17	35.38	0.82	1.26
15	CYP2D6*10/*10	40.98	43.59	1.06	370.14	458.71	1.23	27.01	21.80	0.80	1.21
16	CYP2D6*5/*10	40.45	43.95	1.06	373.15	501.28	1.34	26.79	19.94	0.74	1.36

C_max_: maximum plasma concentration, AUC_0–∞_: area under concentration–time curve from time 0 to infinity, CL: clearance, Obs: observed, Pre: predicted.

**Table 2:** Patients’ demographics and dose-related data of retrieved clinical studies used in PBPK modeling of metoclopramide.

Sr. No.	Reference	Genotype	Dose-Related Information		Demographics			
			N *	Administered Dose (mg)	Age (Years)	Weight (kg)	BMI (Kg/m^2^)	Female Proportion (%)
1	[36]	Non-genotype-specific	6	10	21–24	-	-	83
2		Non-genotype-specific	6	10	69–74	-	-	83
3		Non-genotype-specific	6	10	69–88	-	-	83
4	[37]	Non-genotype-specific	1	10	-	60	-	100
5	[38]	Non-genotype-specific	41	10	19–54	49–108	18–30	33
6	[39]	Non-genotype-specific	18	10	23–29	51–96	-	50
7	[40]	Non-genotype-specific	6	30	21–39	60–95	-	0
8		Non-genotype-specific	6	10	21–39	60–95	-	0
9	[41]	Non-genotype-specific	13	10	18–39	51.5–79.4	18.26–27.47	0
10	[42]	Non-genotype-specific	13	10	20.0–50.0	50.8–91.3	18.01–27.44	0
11	[43]	Non-genotype-specific	14	20	21–29	-	-	0
12	[44]	Non-genotype-specific	8	20	21–37	52–88	-	63
13	[17]	CYP2D6*wt/*wt	13	10	22.5 ± 2.4	64.9 ± 5.4	21.8 ± 1.8	-
14		CYP2D6*wt/*10	9	10	23.0 ± 2.6	63.6 ± 6.3	22.5 ± 1.9	-
15		CYP2D6*10/*10	15	10	22.8 ± 3.4	62.8 ± 6.4	21.9 ± 1.8	-
16		CYP2D6*5/*10	8	10	23.3 ± 3.2	64.5 ± 5.5	22.1 ± 1.6	-

* Number.

**Table 3:** Summarized input data for developing PBPK model of metoclopramide.

Variables	Incorporated Data	Source
**Physicochemical properties**		
Molecular weight (g/mol)	299.79 g/mol	PubChem
Compound type	Basic	PubChem
Plasma protein binding	alpha-1-acid glycoprotein (AAG)	DrugBank
Log P (log unit)	2.00	Adjusted value
pK_a_	9.33, 9.4, 9.27	PubChem
Solubility (mg/mL)	5553.2 µg/mL	[49]
Reference pH	5.5	[49]
**Absorption**		
Intestinal permeability (cm/min)	0.5 × 10^−5^ cm/min	Adjusted value
**Distribution**		
Cellular permeabilities model	PK-Sim standard	-
Partition coefficient mode	Rodgers and Rowland	-
f_u_	0.6	[50]
Specific organ permeability (cm/min)	3.96 × 10^−3^ cm/min	PK-Sim estimated
**Elimination**		
**Km (µM)**		
CYP2D6	53.3	[28]
**V_max_ (pmol/min/pmol)**		
CYP2D6	6.4	[28]
**K_cat_ (L/min)**		
CYP2D6	8.10	Parameter optimization
Total body clearance (L/h/kg)	0.70	[51]
Renal clearance (L/h/kg)	0.16	DrugBank
Specific hepatic clearance (L/h)	1.8	Parameter optimization

Log P: lipophilicity, PK_a_: dissociation constant, f_u_: fraction unbound, CYP: cytochrome P450: K_m_: Michaelis–Menten constant, V_max_: maximal rate of enzymatic reaction, K_cat_: turnover number.

**Table 4:** Enzyme Kinetic Parameters Applied for the Development of the PBPK Model of Metoclopramide.

Sr. No.	Genotype	Activity Scores [52]	Phenotype [13,30]	AS-Based K_cat_	Optimized K_cat_
1	CYP2D6*wt/*wt	2	EM	27.30	27.30
2	CYP2D6*wt/*10	1.25	EM	17.04	13.51
3	CYP2D6*10/*10	0.5	IM	6.82	5.19
4	CYP2D6*5/*10	0.25	IM	3.41	4.05

K_cat_: turnover number.

## Data Availability

The original contributions presented in this study are included in the article/Supplementary Materials. Further inquiries can be directed to the corresponding authors.

## Supplementary Materials

The following supporting information can be downloaded at: https://www.mdpi.com/article/10.3390/ph19071105/s1, Table S1: Output of PK parameters as a result of sensitivity analysis of fraction unbound, specific intestinal permeability, renal plasma clearance, lipophilicity, and solubility at reference pH, specific hepatic clearance, and Kcat; Figure S1. Sensitivity analysis of fraction unbound, specific intestinal permeability, specific renal clearance, lipophilicity, solubility at reference pH, specific hepatic clearance, and Kcat. Kcat: turnover number; Figure S2. Goodness-of-fit plot comparing observed and predicted plasma concentration datasets of metoclopramide. Gray circles, colored circles, solid lines, and dotted lines indicate non-genotype-specific plasma concentrations, genotype-specific plasma concentrations, a line of unity, and a two-fold range, respectively; Figure S3. Residual time plot for the PBPK model of metoclopramide. Gray circles, colored circles, and solid lines indicate residuals for non-genotype-specific studies, residuals for CYP2D6 genotypes (*CYP2D6*wt/*wt*, *CYP2D6*wt/*10*, *CYP2D6*10/*10*, *CYP2D6*5/*10*), and zero line, respectively.

## Abbreviations

The following abbreviations are used in this manuscript:

PBPK	Physiologically based pharmacokinetic
PK	Pharmacokinetic
PD	Pharmacodynamic
VPC	Visual predictive check
R_pre/obs_	Predicted-to-observed ratio
MRD	Mean relative deviation
FDA	Food and Drug Administration
ADME	Absorption, distribution, metabolism, excretion
IVIVE	In vitro–in vivo extrapolation
GIT	Gastrointestinal tract
CTZ	Chemoreceptor trigger zone
UR	Ultrarapid metabolizer
EM	Extensive metabolizer
IM	Intermediate metabolizer
PM	Poor metabolizer
AUC_0–∞_	Area under the concentration–time curve from time 0 to infinity
C_max_	Maximum plasma concentration
CL	Clearance
K_m_	Michaelis–Menten constant
V_max_	Maximal rate of enzymatic reaction
k_cat_	Turnover number
CPIC	Clinical Pharmacogenetics Implementation Consortium
AS	Activity scoring
