# Supplementary material for: Development and Evaluation of Physiologically Based Pharmacokinetic (PBPK) Models to Investigate the Effect of CYP2D6 Polymorphism on Metoclopramide Systemic Exposure
Source: Pharmaceuticals (Basel). 2026 Jul 17;19(7):1105. doi: 10.3390/ph19071105 (PMC13415256; doi:10.3390/ph19071105)
Supplement: Supplementary file 1 [file pharmaceuticals-19-01105-s001.zip › pharmaceuticals-4294265-supplementary.pdf]

Development and Evaluation of Physiologically Based Pharmacokinetic (PBPK) Models to Investigate the Effect of CYP2D6 Polymorphism on Metoclopramide Systemic Exposure

Iqra Shahzad[1], Ammara Zamir[1], Muhammad Fawad Rasool[1*], Amer S. Alali[2], Iltaf Hussain[3], Faleh Alqahtani[4*]

[1]Department of Pharmacy Practice, Faculty of Pharmacy, Bahhaudin Zakariya University, 60800, Multan, Pakistan; rphiqrashahzad0595@gmail.com (I.S) ORCID ID: 0009-0004-1528-9844; ammarazamir20@gmail.com (A.Z) ORCID ID: 0009-0002-8477-3926; fawadrasool@bzu.edu.pk (M.F.R) ORCID ID: 0000-0002-8607-8583

[2]Department of Pharmaceutics, College of Pharmacy, Prince Sattam Bin Abdulaziz University, Al-Kharj 11942, Saudi Arabia; a.alali@psau.edu.sa (A.S.A) ORCID ID: 0000-0003-2057-7918

[3]Center for Drug Safety and Policy, Xi'an Jiatong University, Xi'an PR China; iltafhussain@stu.xjtu.edu.cn (I.H) ORCID ID: 0000-0002-2989-8809

[4]Department of Pharmacology and Toxicology, College of Pharmacy, King Saud University, Riyadh 11451, Saudi Arabia; afaleh@ksu.edu.sa (F.A) ORCID ID: 0000-0003-3924-593X

[*]Corresponding author: Muhammad Fawad Rasool fawadrasool@bzu.edu.pk, Faleh Alqahtani afaleh@ksa.edu.sa

Table S1: Output of PK parameters as a result of sensitivity analysis of fraction unbound, specific intestinal permeability, renal plasma clearance, lipophilicity, and solubility at reference pH, specific hepatic clearance, and K_{cat}

PK Parameter	K_{cat}	F_u	Specific renal clearance	Lipophilicity	Solubility	Specific intestinal permeability	Specific hepatic clearance
% $AUC_{tlast-\infty}$	-0.85	0.84	-0.29	9.13	0	2.31×10^{-4}	-1.08
AUC_{0-inf}	0.18	-0.84	-0.12	-0.48	0	0	-0.41
AUC_{tEnd}	-0.17	-0.86	-0.11	-0.59	0	0	-0.40
C_{max}	-8.28×10^{-4}	-0.69	-0.02	-2.11	0	-5.56×10^{-4}	-1.25
C_{tEnd}	-0.83	-0.22	-0.34	3.89	0	0	-1.25
Half life	-0.20	0.22	-0.07	1.30	0	1.66×10^{-4}	0.24
MRT	-0.24	0.17	-0.09	1.78	0	0	-0.32
t_{max}	0	0	0	0	0	0	0
Cl/F	0.18	0.84	0.12	0.48	0	0	0.41
Vd/F	-0.01	1.07	0.05	1.87	0	2.2×10^{-4}	0.17
Vss/F	-0.06	1.01	0.03	2.36	0	0	0.10

$AUC_{tlast-\infty}$: area under concentration-time curve from last measurable concentration to infinity, AUC_{0-inf}: area under the concentration-time curve from time 0 to infinity, AUC_{tEnd}: area under the concentration-time curve from time 0 to the end of dosing interval, C_{max}: maximum plasma concentration, C_{tEnd}: plasma drug concentration at the end of the dosing interval, MRT: mean residence time, t_{max}: time at maximum plasma concentration, Cl/F: total body clearance/oral bioavailability, Vd/F: volume of distribution/oral bioavailability, Vss/F: volume of distribution at steady state/oral bioavailability, F_u: fraction unbound, K_{cat}: turnover number.

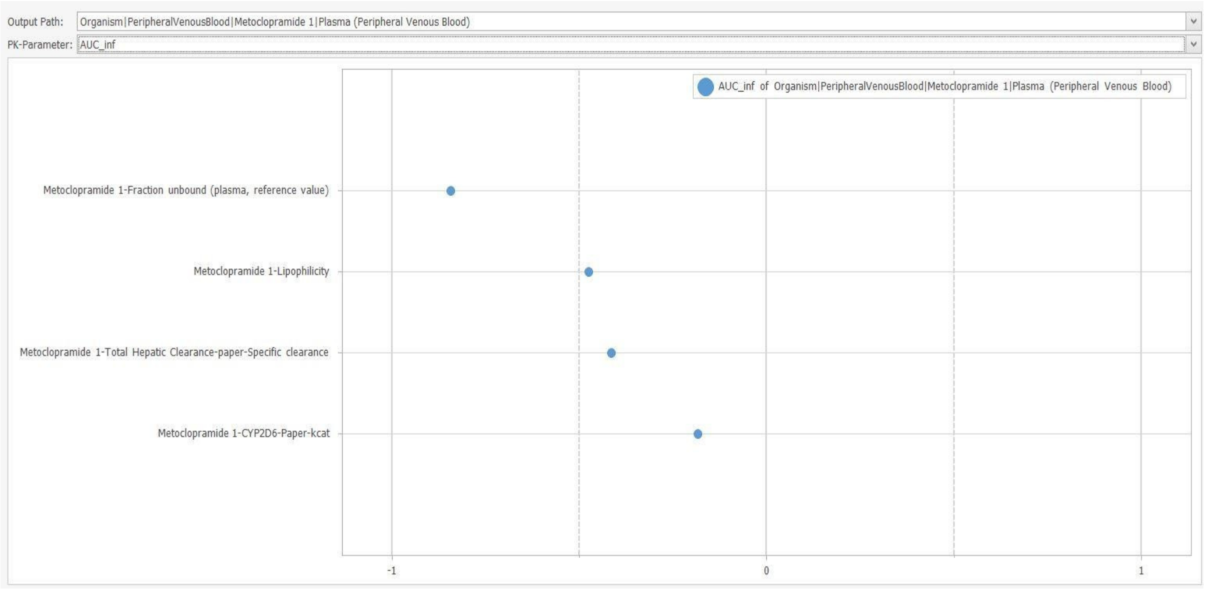

Figure S1. Sensitivity analysis of fraction unbound, specific intestinal permeability, specific renal clearance, lipophilicity, solubility at reference pH, specific hepatic clearance, and $K_{cat.}$ K_{cat}: turnover number

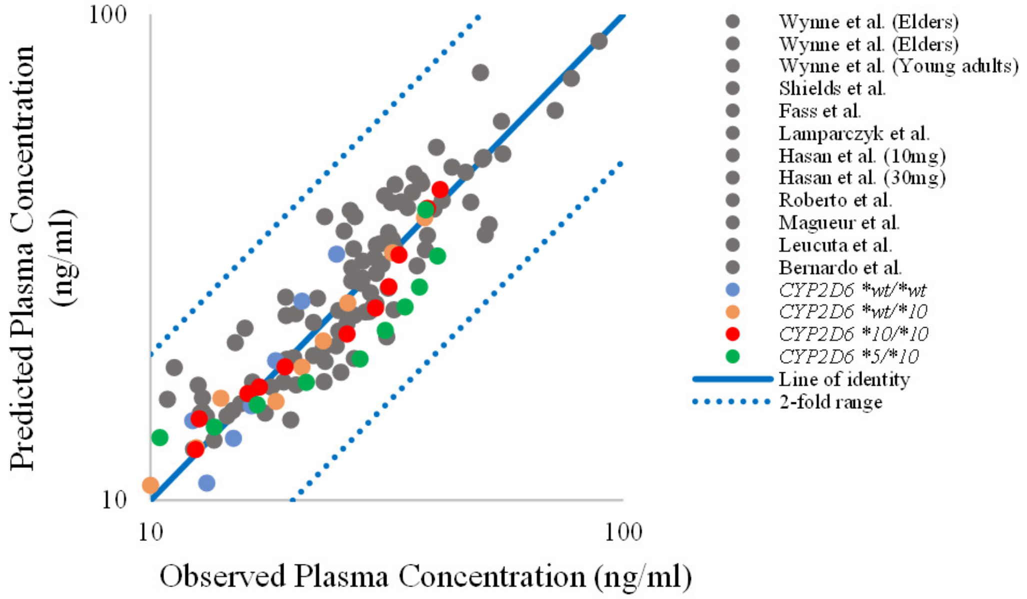

Figure S2. Goodness-of-fit plot comparing observed and predicted plasma concentration datasets of metoclopramide. Gray circles, colored circles, solid lines, and dotted lines indicate non-genotype-specific plasma concentrations, genotype-specific plasma concentrations, a line of unity, and a two-fold range, respectively.

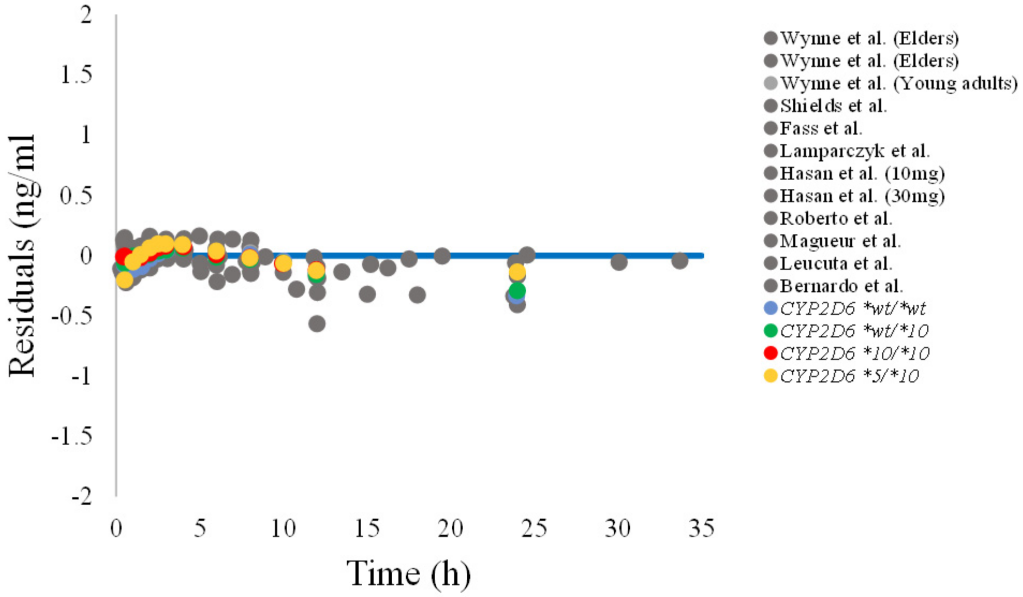

Figure S3. Residual time plot for the PBPK model of metoclopramide. Gray circles, colored circles, and solid lines indicate residuals for non-genotype-specific studies, residuals for CYP2D6 genotypes (*CYP2D6*wt/*wt, CYP2D6*wt/*10, CYP2D6*10/*10, CYP2D6*5/*10),* and zero line, respectively.
